# Comparing self-identified and census-defined neighborhoods among adolescents using GPS and accelerometer

**DOI:** 10.1186/1476-072X-12-57

**Published:** 2013-12-10

**Authors:** Alyssa I Robinson, Nicolas M Oreskovic

**Affiliations:** 1 Center for Child & Adolescent Health Research and Policy, Massachusetts General Hospital, Boston MA, USA; 2 Departments of Internal Medicine and Pediatrics, Massachusetts General Hospital, Boston MA, USA; 3 Harvard Medical School, Boston MA, USA

**Keywords:** Neighborhood, Youth, Physical activity, GIS

## Abstract

**Background:**

Numerous definitions of neighborhood exist, yet few studies have considered youth’s perceptions of neighborhood boundaries. This study compared youth-identified neighborhood (YIN) boundaries to census-defined neighborhood (CDN) boundaries, and determined how the amount of time spent and moderate-to-vigorous physical activity (MVPA) levels compared within both boundary types.

**Methods:**

Adolescents aged 11–14 years were asked to identify their neighborhood boundaries using a map. Objective location and physical activity data collected using Global Positioning System (GPS) devices and accelerometers were used to calculate the amount of time spent and MVPA within youth-identified and census-defined neighborhood boundaries. Paired bivariate analyses compared mean area (meters squared), percent of total time, daily MVPA (minutes), time density (minutes/m^2^) and MVPA density (minutes/m^2^) for both boundary types.

**Results:**

Youth-identified neighborhoods (1,821,705 m^2^) and census-defined neighborhoods (1,277,181 m^2^) were not significantly different in area, p = 0.30. However, subjects spent more time in youth-identified neighborhoods (80.3%) than census-defined neighborhoods (58.4%), p < 0.0001, and engaged in more daily MVPA within youth-identified neighborhoods (14.7 minutes) than census-defined neighborhoods (9.5 minutes), p < 0.0001. After adjusting for boundary area, MVPA density (minutes of MVPA per squared meter of area) remained significantly greater for youth-identified neighborhoods (2.4 × 10^-4^ minutes/m^2^) than census-defined neighborhoods (1.4 × 10^-4^ minutes/m^2^), p = 0.02.

**Conclusions:**

Adolescents perceive their neighborhoods to be similar in size to census-defined neighborhoods. However, youth-identified neighborhoods better capture the locations in which adolescents spend time and engage in physical activity. Asking adolescents to identify their neighborhood boundaries is a feasible and valuable method for identifying the spaces that adolescents are exposed to and use to be physically active.

## Background

Environmental factors are thought to play an important role in child and adolescent health. A policy statement on the built environment from the American Academy of Pediatrics Committee on Environmental Health recognized the ability of the environment to affect children’s health and lives [1]. Not surprisingly, within public health research there has been a heightened interest in how both perceived and objective environmental attributes influence youth’s behavior patterns and health outcomes, including food purchasing behaviors [2], obesity [3-5], and physical activity [6-8]. Efforts have been made to establish standardized methods of quantifying various place characteristics, such as greenness, food environment, mixed land use, and street connectivity. However, there is little consensus on how best to identify and operationalize relevant areas of exposure.

Researchers have recognized the importance of accurately defining neighborhood and the challenges in doing so. Historically, place effects research has relied on neighborhood proxies including census and administrative boundaries [5,9], predefined Euclidean and street network buffers around the home or school area [2,6-8,10-13], and resident report of easy walking or driving distance [14,15]. Recent reviews of the environmental effects literature found an overwhelming dependence on administrative units [16-18], a trend also reflected in adolescent physical activity research [19-21]. While using census boundaries and predetermined buffers may be a convenient means of approximating neighborhood, these proxies are inadequate in identifying the various spaces that individuals travel to and are exposed to and thereby the factors that may impact health [22-25].

One challenge in studying place effects on health and behavior arises from the *uncertain geographic context problem (UGCoP)*, a lack of certainty as to which geographic contexts truly influence an individual [25]. As a result, researchers have emphasized looking beyond proximate, residential neighborhoods to consider the multiplicity of activity spaces that an individual is exposed to on a day-to-day basis [24,26,27]. More recently, research has explored new ways to better capture and think about the spaces in which individuals spend their time, ranging from the use of Global Positioning System (GPS) devices [28-33] to consideration of varying levels of neighborhood aggregation [34]. Another approach has been to account for residents’ perceptions in conceptualizing neighborhood. Studies looking at adults’ views of neighborhood boundaries have found discrepancies between resident perceptions of neighborhood and census tracts [35] or buffers [14]. Different variations of cognitive mapping have also been used in youth. One study asked children to draw and photograph their home and neighborhood environments, but only identified qualitative neighborhood themes, rather than a measurable neighborhood area [36]. Others have utilized mapping to identify children’s self reported travel to play spaces [37] and activity space [27] as well as compare hand drawn activity paths and neighborhoods to administrative boundaries [38]. Especially with regard to youth populations, there is limited research on the efficacy of these methodologies and little consensus regarding the most appropriate way to identify exposure areas in order to measure their effect.

To date, few studies have attempted to account for adolescents’ perceptions of neighborhood and to our knowledge none have assessed to what extent objectively measured movement and physical activity patterns are reflective of their perceptions of neighborhood boundaries versus census-defined boundaries, such as census tracts. The aims of this study were to i) test the feasibility of asking adolescents to identify their neighborhoods, ii) compare youth-identified neighborhood (YIN) boundaries to census-defined neighborhood (CDN) boundaries, and iii) determine how the amount of time spent and moderate-to-vigorous physical activity (MVPA) levels compare in youth-identified and census-defined neighborhoods. Though this study explored the delineation of neighborhood boundaries, our intention was not to limit the notion of neighborhood to solely residential spaces. Rather, our aim was to broaden the scope of what constitutes neighborhood by additionally including non-residential activity spaces.

## Methods

### Participants

We recruited 32 non-Hispanic white, non-Hispanic black, and Hispanic adolescents aged 11–14 years who were residents of three towns located in the greater Boston, Massachusetts area. Subjects were recruited from a local community health center and a community recreation center. Informed consent along with child assent were obtained from each family prior to participation. This study was approved by the Partners HealthCare Institutional Review Board.

### Location and physical activity data collection and measures

Each subject was asked to wear an elastic belt around the hip equipped with a GPS receiving unit (QStarz BT-Q1000XT) to record location and an accelerometer (GT3X; ActiGraph LLC) to record physical activity. Both devices were set to record in 30 second epochs, with their internal clocks synchronized to the Universal Time Clock. Subjects were asked to wear the belt for two separate weeks (5 weekdays and 2 weekend days) between May 2011 and May 2012 and were instructed to wear the belt at all times except during water activities (e.g. bathing, swimming, etc.) and sleep hours. To account for seasonality, GPS and accelerometer data were collected during both a warm and cold season. Participants were given a charger for the GPS unit and instructed to charge the device overnight. Self-reported age (date of birth), sex, and race/ethnicity were obtained along with home and school address.

### Neighborhood map design

Maps (17″ × 22″) were created in ArcMap 10 (ESRI, Redlands, Calif) using the ESRI World Street Map basemap layer (updated July 2012) at a zoom scale of 1:22,000. The basemap contained major and minor roads, highways, railways, water features, administrative boundaries, cities, parks, and landmarks [39]. This map size and zoom were selected to achieve adequate street and landmark detail in addition to some geographic breadth. Each subject’s map included his/her town of residence and bordering towns. Massachusetts’ Town Boundaries, MBTA Rapid Transit Lines and Stations, and Schools (PK-High School) were mapped for orientation purposes using data from MassGIS [40]*.* Each subject’s home and school were geocoded and also identified on the map.

### Neighborhood map data collection and measures

Each subject met individually with a trained research staff member between October 2012 and January 2013 and was provided with a map and instructions which were reviewed aloud. Subjects were asked to outline on the map the area(s) that they considered to be part of their neighborhood and were told that they could outline one or several area(s). Subjects were instructed to completely enclose all outlined areas and were assured that there was no single right or wrong answer. Subjects were told to inquire with research staff if there was an area that they wished to include, but could not locate on the map. Neighborhood was defined for subjects as “the area(s) in which you live and where you spend your time”.

### Data processing

#### Data merging and processing

Each subject’s GPS and accelerometer output files were manually reviewed by study personnel upon return to ensure that both output files contained adequate data for analysis. The two files were joined and location and physical activity data were matched based on date and time. Joined datasets were validated and cleaned using a multistep approach: 1) a valid hour of combined data was required to have a minimum of 10% non-zero accelerometer epochs with matching GPS datapoints, 2) a valid day of combined data was required to have at least 2 valid hours, 3) a valid dataset was required to have at least 2 valid weekdays and 1 valid weekend day of combined data. Datasets that met the minimum inclusion criteria were then cleaned to exclude days and/or hours of non-wear as defined by the validation criteria above using steps 1 and 2. For instances in which any accelerometer datapoint did not have a corresponding GPS point, the missing latitude and longitude were imputed using the last previously recorded location. In order to avoid imputation errors, datasets were manually inspected and data were removed when there was i) imputation of GPS data from a prior day (to avoid day cross over) or ii) prolonged missing GPS data (>2 consecutive hours) during non-school hours. These criteria were chosen to avoid potential erroneous imputation during non-school hours, as we could not ensure that prolonged periods of consecutive missing GPS data were due to indoor signal loss, rather than GPS malfunction or battery depletion. The joined data were collapsed into 1 minute epochs and overnight hours (12 am-5 am) were removed from analyses.

#### Physical activity classifications

Accelerometer data were classified into two intensity categories: moderate-to-vigorous physical activity (MVPA) (≥2296 counts-per-minute, CPM) and non MVPA (<2296 CPM), based on age-appropriate cut-points [41,42].

#### GIS data processing

**Figure 1:**
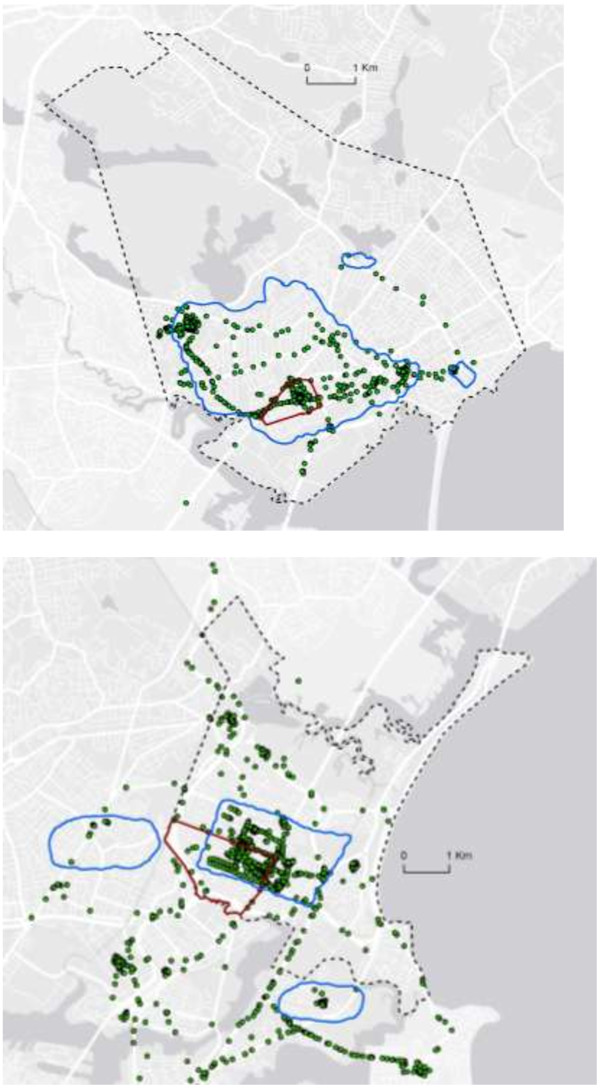
**Example of GIS maps of neighborhood boundaries and combined GPS and accelerometer datapoints.** Figure Legend: Census-Defined Neighborhood boundaries (outlined in red), Youth-Identified Neighborhood boundaries (outlined in blue) and combined location and physical activity data (green points).

Each subject’s completed map was scanned, imported into ArcMap, and georeferenced. Youth-identified neighborhood (YIN) outlines were manually traced to create neighborhood polygons. Census-defined neighborhood (CDN) polygons were created for each subject based on his/her census tract using Massachusetts census data obtained from MassGIS. Bodies of water were excluded from both subject polygons and census tract polygons. Area (in meters squared) was calculated for each subject’s YIN and CDN boundaries in addition to the number of combined GPS and accelerometer datapoints that fell within each subject’s two boundary types (Figure 1).

### Analysis

Descriptive statistics were used to calculate the area in meters squared of census-defined and youth-identified neighborhoods, the percent overlap between CDN and YIN (% of YIN area that fell within CDN boundaries), the mean daily minutes of MVPA in CDN and YIN, and the percent of total time spent in CDN and YIN. To adjust for boundary area and measure the distribution of time spent and MVPA across the two boundary types, time density (minutes/m^2^) and MVPA density (minutes/m^2^) were calculated by dividing subjects’ total time spent and total minutes of MVPA within CDN and YIN by their respective boundary areas. Paired bivariate analyses using non-parametric Wilcoxon tests compared mean areas for YIN and CDN boundaries, mean daily minutes of MVPA, and mean percent of total time per boundary type, as well as mean MVPA density and time density per boundary type.

## Results

Thirty-two subjects were invited and agreed to participate in the study. One subject had difficulty following study instructions and inspection of the neighborhood map revealed that the data provided were not adequate for interpretation and the subject was therefore excluded from further analyses. The mean age of the remaining thirty-one subjects was 12 years, with 9 (29%) male, 14 (45%) white, 6 (19%) black, 11 (35%) Hispanic or Latino. Mean daily minutes of MVPA per subject was 21.1 (SD 11.7). On average, subjects provided 389.5 minutes (range: 178.8-600.6) of raw, matching GPS and accelerometer data per day. After imputation of missing location data, subjects had an average of 656.6 minutes (range: 504.2-779.8) of combined GPS and accelerometer data per day.

Twenty seven subjects outlined multiple noncontiguous areas to represent their neighborhoods. Thirty subjects identified areas that fell at least partly outside of their census-defined neighborhood (Figure). On average, 31.2% of subjects’ self-identified neighborhood area fell within their census tract boundaries (range: 4.7%-87.6%). Of the 31 subjects’ neighborhood boundaries, 29 YIN boundaries included subjects’ school locations, whereas only 7 CDN did so.

The results of the neighborhoods’ descriptive characteristics are presented in the Table 1. The mean area for YIN (1,821,705 m^2^) was not significantly different than the mean area for CDN (1,277,181 m^2^), p = 0.30. Despite being comparable in size, adolescents spent significantly more of their overall time within self-identified neighborhood boundaries (80.3%) than census-defined boundaries (58.4%), p < 0.0001. On average, adolescents also engaged in more daily MVPA within YIN (14.7 minutes) than CDN (9.8 minutes), p < 0.0001. After adjusting for boundary area, there were no differences in time density for youth-identified (5.7 × 10^-3^ minutes/m^2^) and census-defined neighborhoods (8.3 × 10^-3^ minutes/m^2^), p = 0.19, though MVPA density was significantly greater for YIN (2.4 × 10^-4^ minutes/m^2^) than CDN (1.4 × 10^-4^ minutes/m^2^) boundaries, p = 0.02.

**Table 1:** Census-Defined Neighborhood (CDN) and Youth-Identified Neighborhood (YIN) characteristics

	**CDN**	**YIN**	***p*-value**
Area (m^2^)	1,277,181	1,821,705	0.30
Percent of daily time	58.4	80.3	<0.0001
Daily MVPA (minutes)	9.5	14.7	<0.0001
Time density (minutes/m^2^)	5.7x10^-3^	8.0x10^-3^	0.19
MVPA density (minutes/m^2^)	1.4x10^-4^	2.4x10^-4^	0.02

MVPA = moderate-to-vigorous physical activity.

## Discussion

Despite increasing interest in how youth’s environments affect their health, there remains little agreement as to how best to operationalize and measure exposure areas in this population. This study tested the feasibility of asking adolescents to identify what they perceived to be their residential neighborhood and activity spaces and looked at how youth-identified neighborhood boundaries compare to census tract boundaries, which are commonly used in studying neighborhood effects. We found this to be not only feasible, but a valuable, novel method for identifying neighborhood boundaries in this age group.

Though past research on neighborhood environments has traditionally focused on residential neighborhoods, the current study aimed to expand the notion of neighborhood to better incorporate the multiplicity of spaces in which adolescents spend time. It is well recognized that daily activities frequently occur outside of residential neighborhoods and that shifting focus to activity spaces may allow for a more thorough understanding of environmental exposures [24,26,43]. We aimed to consider both residential neighborhoods and non-residential activity spaces concurrently by prompting adolescents to identify the spaces in which they live and where they spend time.

Our results suggest that youth-identified neighborhood area(s) are similar in size to census tracts. However, unlike census-defined neighborhoods which consist of a single administrative unit (the census tract), a majority of youth-identified neighborhoods consisted of multiple noncontiguous areas. A similar study which asked late adolescent males to identify their neighborhoods also found some evidence of this pattern [38]. Though not surprising, this finding emphasizes the inadequacy of using a single discrete geographic unit as a proxy for neighborhood, or exposure area more generally. The observation that subjects spent time within multiple ‘neighborhood areas’ is consistent with the notions of spatial polygamy and polycentric exposures described by Matthews and Yang [44] as well as theories of time-space geography [45]. For adolescents in particular, these patterns may be due to practical and social constraints such as dependency on adults as both a source of transportation and authority. Furthermore, almost all subjects outlined areas that fell outside of their census tract and several subjects identified areas outside of their town of residence. Census tracts have an extensive history of use in research on place effects on health and behavior [16-18], including youth physical activity [19-21].

In addition, we found that youth-identified neighborhoods captured significantly more of adolescents’ overall time and daily MVPA than census-defined neighborhoods, suggesting that youth-identified boundaries may serve as a better proxy for exposure area than census tracts in studying adolescent physical activity and risk exposures. Not only are adolescents’ self-identified boundaries capable of capturing exposure areas that census tract neighborhoods cannot, but they appear to do so accurately. We examined this further by calculating time density and MVPA density to look at the distribution of time spent and MVPA across neighborhood area, measuring the minutes of time spent and minutes of MVPA within each boundary type per squared meter. These additional density measures are important as area-unadjusted measures of time and physical activity do not account for the possibility that larger boundaries may overestimate the neighborhood area while capturing the locations where a subject spends time or is physically active. We found that MVPA density was greater for YIN than CDN, though time density was not different for either boundary type. This suggests that YIN boundaries capture more of adolescents’ daily MVPA than CDN boundaries, with a greater degree of specificity. While YIN boundaries also capture more overall time than CDN, they are comparable to CDN in the distribution of time captured across neighborhood area.

This is one of few studies to ask adolescents to identify their neighborhood boundaries in this manner. Cognitive mapping has been used with children and adolescents in past research, but infrequently to identify the geographic boundaries of their neighborhoods or activity spaces. Furthermore, many studies on environment effects focus only on a single context (e.g. school, home, work, etc.), while adults and youth alike exist within several contexts [22]. We aimed to define neighborhood in a way that encompasses the various contexts in which adolescents function as perceived by adolescents themselves, rather than selectively focusing on a single context such as school or home.

Objective location and physical activity data coupled with the use of GIS mapping techniques allowed us to measure the extent to which movement and MVPA patterns in adolescents are reflective of perceived neighborhood boundaries versus census tract boundaries. The use of GPS and accelerometer data to compliment subjects’ neighborhood maps is a novel component of this study and provides an objective comparison of the effectiveness of these two definitions of neighborhood. Though still relatively new technologies, GPS devices and accelerometers have been found to be valid methods of assessing travel patterns and physical activity, respectively [46-52].

This study has several limitations. Though the use of objective location data is a strength of this study, one disadvantage of GPS receivers is the potential for indoor satellite signal loss. School-age youth spend a significant amount of time indoors (e.g. home, school, etc.), and including the data collected during these indoor times is important to present a complete picture of where adolescents spend their time throughout the day and the environments they are exposed to. To capture this data, we chose to impute missing location rather than lose a large portion of adolescents’ waking hours. Though the potential for imputation of incorrect location data exists if battery depletion or GPS device malfunction were misinterpreted to be indoor activity, we took several precautions to avoid such errors, including exclusion of imputed data from a previous day and prolonged consecutive periods of missing data during non-school hours.

The inclusion of school data may partly explain our study results. We found that approximately three-quarters of census-defined neighborhoods excluded subjects’ school locations. Given that adolescents spend a significant amount of waking hours at school, it is possible that the difference we observed in overall time spent is a result of the inclusion or exclusion of school location within neighborhood boundaries. This further highlights an additional shortcoming of traditional methods of identifying neighborhoods, which may provide a biased and incomplete picture of where youth spend time throughout the day. Given these findings, one practical consideration in identifying exposure areas may be to include both school and home area.

Though we found that YIN boundaries captured more overall time and MVPA than CDN boundaries, the current study did not explore the time spent and MVPA outside of both boundary areas. Future research may wish to consider potential differences between CDN and YIN environments and those environments not captured by either boundary type. One possibility is that adolescents (and census boundaries) do not account for the journeys from place to place, which are likely a significant source of exposure time and MVPA [53].

Our study instructions defined neighborhood as the location(s) where subjects lived in addition to where they spent their time, potentially influencing how subjects perceived and drew their neighborhoods. This definition of neighborhood was chosen to incorporate both residential neighborhood, which alone has proven inadequate in assessing environmental exposures, and activity spaces. Additionally, a previous study using similar methodology found that when asked to draw their neighborhood on a map without further instruction, subjects interpreted neighborhood to mean where they spent their time [38]. This also suggests that while place effects researchers distinguish between ‘neighborhood’ (i.e. residential neighborhood) and activity space, adolescents may not.

It is possible that subjects chose to include the areas that they could easily locate, although subjects were instructed to request assistance from research staff if they had difficulty finding an area on the map. Another limitation of this study may be a lack of generalizability of the data due to both the small sample size and limited age range and the potential for bias resulting from the socially homogenous sample (all recruited from community health center and recreation center). Similarly, it may be difficult to group individuals by neighborhood area using unique and resident-specific boundaries. Asking subjects to identify their neighborhoods using a GIS map may require a certain amount of geographic awareness, and likely has a lower age limit. Research has also suggested possible differences among urban and rural populations [15,28].

Future studies using these methods would benefit from a larger sample size and should examine potential differences by age, gender, race/ethnicity, and weather. In this study, we conducted the neighborhood mapping activity only once, though others may aim to assess test-retest reliability. We also chose census boundaries as our comparison measure, as they have a long history of use in neighborhood effects and health research. However, an alternative approach may be to compare youth-identified neighborhoods to other neighborhood proxies such as buffers, which are being used more frequently in defining neighborhoods especially in youth populations. As GIS technology advances, place effects research will benefit from new opportunities to explore exposure areas. One improvement on the methodology in this study could be the use of a more interactive mapping activity, such as that used by Chaix and colleagues (2012) [54], in which subjects are able to manipulate the map scale and therefore view the map at varying degrees of detail. Additionally, future studies may use GIS to examine potential differences in the built environments of CDN and YIN areas.

These results suggest that there may be feasible alternatives to using the existing operational definitions of neighborhood, which has implications for health researchers, urban planners, and policymakers. Though the use of census data and other common proxies may be convenient, the detriments of misattributing environmental variables to health outcomes and behavioral patterns and using this information to shape urban policy and planning may outweigh the benefits of convenience. Being able to better capture adolescents’ residential neighborhoods and activity spaces could allow for better identification of the factors that affect adolescents’ health and in particular their physical activity patterns. Furthermore, the capacity to better identify the spaces used by adolescents could allow policymakers and urban planners to more appropriately allocate resources and target changes in the environment. Considering the increasing interest in how environments affect health outcomes in children and adolescents, further investigation into better ways to identify exposure areas are needed, especially in youth.

## Conclusions

Asking adolescents to identify the boundaries of their neighborhood is a feasible and innovative method for identifying and measuring exposure areas. Youth-identified neighborhoods are not significantly different in size from census tract boundaries, but appear to better capture the locations in which adolescents spend their time and engage in physical activity.

## Abbreviations

YIN: Youth-identified neighborhood; CDN: Census-defined neighborhood; MVPA: Moderate-to-vigorous physical activity; GPS: Global positioning system.

## Competing interests

The authors declare that they have no competing interests.

## Authors’ contributions

AR and NO contributed to the design of the study and data analysis. AR collected the data and drafted the manuscript under the guidance of NO. NO made important contributions to several sections and reviewed the entire manuscript. Both authors read and approved the final manuscript.

## References

[B1] Tester JM Committee on Environmental Health The built environment: designing communities to promote physical activity in children Pediatrics 2009 12 6 1591 1598 19482771 10.1542/peds.2009-0750

[B2] He M Tucker P Gilliland J Irwin JD Larsen K Hess P The influence of local food environments on adolescents’ food purchasing behaviors Int J Environ Res Public Health 2012 12 4 1458 1471 22690205 10.3390/ijerph9041458 PMC3366623

[B3] Burdette HL Whitaker RC A national study of neighborhood safety, outdoor play, television viewing, and obesity in preschool children Pediatrics 2005 12 3 657 662 10.1542/peds.2004-2443 16140705

[B4] Nogueira H Ferrao M Gama A Mourao I Rosado Marques V Padez C Perceptions of neighborhood environments and childhood obesity: evidence of harmful gender inequities among Portuguese children Health Place 2013 12 69 73 23201911 10.1016/j.healthplace.2012.10.005

[B5] Saelens BE Sallis JF Frank LD Couch SC Zhou C Colburn T Cain KL Chapman J Glanz K Obesogenic neighborhood environments, child and parent obesity: the neighborhood impact on kids study Am J Prev Med 2012 12 5 e57 e64 10.1016/j.amepre.2012.02.008 22516504 PMC3332077

[B6] Prins RG Oenema A van der Horst K Brug J Objective and perceived availability of physical activity opportunities: differences in associations with physical activity behavior among urban adolescents Int J Behav Nutr Phys Act 2009 12 70 10.1186/1479-5868-6-70 19832969 PMC2770555

[B7] Almanza E Jerrett M Dunton G Seto E Pentz MA A study of community design, greenness, and physical activity in children using satellite, GPS and accelerometer data Health Place 2012 12 1 46 54 10.1016/j.healthplace.2011.09.003 22243906 PMC3399710

[B8] Cohen DA Ashwood JS Scott MM Overton A Evenson KR Staten LK Porter D McKenzie TL Catellier D Public parks and physical activity among adolescent girls Pediatrics 2006 12 5 e1381 e1389 10.1542/peds.2006-1226 17079539 PMC2239262

[B9] Hackman DA Betancourt LM Brodsky NL Hurt H Farah MJ Neighborhood disadvantage and adolescent stress reactivity Front Hum Neurosci 2012 12 277 23091454 10.3389/fnhum.2012.00277 PMC3469875

[B10] Duncan DT Castro MC Gortmaker SL Aldstadt J Melly SJ Bennett GG Racial differences in the built environment--body mass index relationship? A geospatial analysis of adolescents in urban neighborhoods Int J Health Geogr 2012 12 11 10.1186/1476-072X-11-11 22537116 PMC3488969

[B11] Wilson DK Lawman HG Segal M Chappell S Neighborhood and parental supports for physical activity in minority adolescents Am J Prev Med 2011 12 4 399 406 10.1016/j.amepre.2011.06.037 21961467 PMC3278802

[B12] Pate RR Colabianchi N Porter D Almeida MJ Lobelo F Dowda M Physical activity and neighborhood resources in high school girls Am J Prev Med 2008 12 5 413 419 10.1016/j.amepre.2007.12.026 18407008 PMC2408745

[B13] Wolch J Jerrett M Reynolds K McConnell R Chang R Dahmann N Brady K Gilliland F Su JG Berhane K Childhood obesity and proximity to urban parks and recreational resources: a longitudinal cohort study Health Place 2011 12 1 207 214 10.1016/j.healthplace.2010.10.001 21075670 PMC4380517

[B14] Smith G Gidlow C Davey R Foster C What is my walking neighbourhood? A pilot study of English adults’ definitions of their local walking neighbourhoods Int J Behav Nutr Phys Act 2010 12 34 10.1186/1479-5868-7-34 20459636 PMC2873577

[B15] Colabianchi N Dowda M Pfeiffer KA Porter DE Almeida MJ Pate RR Towards an understanding of salient neighborhood boundaries: adolescent reports of an easy walking distance and convenient driving distance Int J Behav Nutr Phys Act 2007 12 66 10.1186/1479-5868-4-66 18088416 PMC2225417

[B16] Leal C Chaix B The influence of geographic life environments on cardiometabolic risk factors: a systematic review, a methodological assessment and a research agenda Obes Rev 2011 12 3 217 230 10.1111/j.1467-789X.2010.00726.x 20202135

[B17] Feng J Glass TA Curriero FC Stewart WF Schwartz BS The built environment and obesity: a systematic review of the epidemiologic evidence Health Place 2010 12 2 175 190 10.1016/j.healthplace.2009.09.008 19880341

[B18] Riva M Gauvin L Barnett TA Toward the next generation of research into small area effects on health: a synthesis of multilevel investigations published since July 1998 J Epidemiol Community Health 2007 12 10 853 861 10.1136/jech.2006.050740 17873220 PMC2652961

[B19] Kim J Liu J Colabianchi N Pate RR The effect of perceived and structural neighborhood conditions on adolescents’ physical activity and sedentary behaviors Arch Pediatr Adolesc Med 2010 12 10 935 942 20921351 10.1001/archpediatrics.2010.167 PMC4410858

[B20] Carroll-Scott A Gilstad-Hayden K Rosenthal L Peters SM McCaslin C Joyce R Ickovics JR Disentangling neighborhood contextual associations with child body mass index, diet, and physical activity: the role of built, socioeconomic, and social environments Soc Sci Med 2013 12 106 114 23642646 10.1016/j.socscimed.2013.04.003 PMC4058500

[B21] Zhang X Christoffel KK Mason M Liu L Identification of contrastive and comparable school neighborhoods for childhood obesity and physical activity research Int J Health Geogr 2006 12 14 10.1186/1476-072X-5-14 16573835 PMC1526711

[B22] Ball K Timperio AF Crawford DA Understanding environmental influences on nutrition and physical activity behaviors: where should we look and what should we count? Int J Behav Nutr Phys Act 2006 12 33 10.1186/1479-5868-3-33 16999874 PMC1592115

[B23] Flowerdew R Manley DJ Sabel CE Neighbourhood effects on health: does it matter where you draw the boundaries? Soc Sci Med 2008 12 6 1241 1255 10.1016/j.socscimed.2007.11.042 18177988

[B24] Chaix B Merlo J Evans D Leal C Havard S Neighbourhoods in eco-epidemiologic research: delimiting personal exposure areas: a response to Riva, Gauvin, Apparicio and Brodeur Soc Sci Med 2009 12 9 1306 1310 10.1016/j.socscimed.2009.07.018 19692161

[B25] Kwan MP The uncertain geographic context problem Ann Assoc Am Geogr 2012 12 5 958 968 10.1080/00045608.2012.687349

[B26] Cummins S Curtis S Diez-Roux AV Macintyre S Understanding and representing ‘place’ in health research: a relational approach Soc Sci Med 2007 12 9 1825 1838 10.1016/j.socscimed.2007.05.036 17706331

[B27] Villanueva K Giles-Corti B Bulsara M McCormack GR Timperio A Middleton N Beesley B Trapp G How far do children travel from their homes? Exploring children’s activity spaces in their neighborhood Health Place 2012 12 2 263 273 10.1016/j.healthplace.2011.09.019 22001753

[B28] Boruff BJ Nathan A Nijenstein S Using GPS technology to (re)-examine operational definitions of ‘neighbourhood’ in place-based health research Int J Health Geogr 2012 12 22 10.1186/1476-072X-11-22 22738807 PMC3490929

[B29] Oreskovic NM Blossom J Field AE Chiang SR Winickoff JP Kleinman RE Combining global positioning system and accelerometer data to determine the locations of physical activity in children Geospat Health 2012 12 2 263 272 22639128 10.4081/gh.2012.144

[B30] Wiehe SE Carroll AE Liu GC Haberkorn KL Hoch SC Wilson JS Fortenberry JD Using GPS-enabled cell phones to track the travel patterns of adolescents Int J Health Geogr 2008 12 22 10.1186/1476-072X-7-22 18495025 PMC2426678

[B31] Chaix B Meline J Duncan S Merrien C Karusisi N Perchoux C Lewin A Labadi K Kestens Y GPS tracking in neighborhood and health studies: a step forward for environmental exposure assessment, a step backward for causal inference? Health Place 2013 12 46 51 23425661 10.1016/j.healthplace.2013.01.003

[B32] Kerr J Duncan S Schipperijn J Using global positioning systems in health research: a practical approach to data collection and processing Am J Prev Med 2011 12 5 532 540 10.1016/j.amepre.2011.07.017 22011426

[B33] Krenn PJ Titze S Oja P Jones A Ogilvie D Use of global positioning systems to study physical activity and the environment: a systematic review Am J Prev Med 2011 12 5 508 515 10.1016/j.amepre.2011.06.046 22011423 PMC3821057

[B34] Messer LC Vinikoor-Imler LC Laraia BA Conceptualizing neighborhood space: consistency and variation of associations for neighborhood factors and pregnancy health across multiple neighborhood units Health Place 2012 12 4 805 813 10.1016/j.healthplace.2012.03.012 22551891 PMC3856318

[B35] Coulton CJ Korbin J Chan T Su M Mapping residents’ perceptions of neighborhood boundaries: a methodological note Am J Community Psychol 2001 12 2 371 383 10.1023/A:1010303419034 11446289

[B36] Hume C Salmon J Ball K Children’s perceptions of their home and neighborhood environments, and their association with objectively measured physical activity: a qualitative and quantitative study Health Educ Res 2005 12 1 1 13 15253992 10.1093/her/cyg095

[B37] Veitch J Salmon J Ball K Children’s active free play in local neighborhoods: a behavioral mapping study Health Educ Res 2008 12 5 870 879 18039726 10.1093/her/cym074

[B38] Basta LA Richmond TS Wiebe DJ Neighborhoods, daily activities, and measuring health risks experienced in urban environments Soc Sci Med 2010 12 11 1943 1950 10.1016/j.socscimed.2010.09.008 20980088 PMC2982925

[B39] ArcGIS online map and geoservices http://www.esri.com/software/arcgis/arcgisonline/maps/maps-and-map-layers

[B40] Office of geographic information (MassGIS) http://www.mass.gov/mgis/

[B41] Evenson KR Catellier DJ Gill K Ondrak KS McMurray RG Calibration of two objective measures of physical activity for children J Sports Sci 2008 12 14 1557 1565 10.1080/02640410802334196 18949660

[B42] Trost SG Loprinzi PD Moore R Pfeiffer KA Comparison of accelerometer cut points for predicting activity intensity in youth Med Sci Sports Exerc 2011 12 7 1360 1368 10.1249/MSS.0b013e318206476e 21131873

[B43] Zenk SN Schulz AJ Matthews SA Odoms-Young A Wilbur J Wegrzyn L Gibbs K Braunschweig C Stokes C Activity space environment and dietary and physical activity behaviors: a pilot study Health Place 2011 12 5 1150 1161 10.1016/j.healthplace.2011.05.001 21696995 PMC3224849

[B44] Matthews SAYT Spatial Polygamy and Contextual Exposures (SPACEs): promoting activity space approaches in research on place and health Am Behav Sci 2013 12 8 1057 1081 10.1177/0002764213487345 24707055 PMC3975622

[B45] Hägerstrand T Innovation diffusion as a spatial process 1967 Chicago, IL: University of Chicago Press

[B46] Rodriguez DA Brown AL Troped PJ Portable global positioning units to complement accelerometry-based physical activity monitors Med Sci Sports Exerc 2005 12 11 Suppl S572 S581 16294120 10.1249/01.mss.0000185297.72328.ce

[B47] Maddison R Ni Mhurchu C Global positioning system: a new opportunity in physical activity measurement Int J Behav Nutr Phys Act 2009 12 73 10.1186/1479-5868-6-73 19887012 PMC2777117

[B48] Duncan MJ Mummery WK Dascombe BJ Utility of global positioning system to measure active transport in urban areas Med Sci Sports Exerc 2007 12 10 1851 1857 10.1249/mss.0b013e31811ff31e 17909415

[B49] Duncan MJ Badland HM Mummery WK Applying GPS to enhance understanding of transport-related physical activity J Sci Med Sport 2009 12 5 549 556 10.1016/j.jsams.2008.10.010 19237315

[B50] Oliver M Badland H Mavoa S Duncan MJ Duncan S Combining GPS, GIS, and accelerometry: methodological issues in the assessment of location and intensity of travel behaviors J Phys Act Health 2010 12 1 102 108 20231761 10.1123/jpah.7.1.102

[B51] Wheeler BW Cooper AR Page AS Jago R Greenspace and children’s physical activity: a GPS/GIS analysis of the PEACH project Prev Med 2010 12 2 148 152 10.1016/j.ypmed.2010.06.001 20542493

[B52] Troped PJ Wilson JS Matthews CE Cromley EK Melly SJ The built environment and location-based physical activity Am J Prev Med 2010 12 4 429 438 10.1016/j.amepre.2009.12.032 20307812 PMC3568665

[B53] Rainham DG Bates CJ Blanchard CM Dummer TJ Kirk SF Shearer CL Spatial classification of youth physical activity patterns Am J Prev Med 2012 12 5 e87 e96 10.1016/j.amepre.2012.02.011 22516507

[B54] Chaix B Kestens Y Perchoux C Karusisi N Merlo J Labadi K An interactive mapping tool to assess individual mobility patterns in neighborhood studies Am J Prev Med 2012 12 4 440 450 10.1016/j.amepre.2012.06.026 22992364

